# Modern homophobia among heterosexual Romanian adults: the roles of sexual orientation beliefs, religiosity, perceived social roles, and social media use

**DOI:** 10.3389/fpsyg.2023.1219442

**Published:** 2023-09-22

**Authors:** Georgiana Lăzărescu, Adina Karner-Hutuleac, Alexandra Maftei

**Affiliations:** Faculty of Psychology and Education Sciences, Alexandru Ioan Cuza University, Iași, Romania

**Keywords:** homophobia, religiosity, sexual orientation beliefs, gender, time spent online

## Abstract

The present study aimed to examine some potential predictors of homophobia against lesbians and gay individuals. Our sample comprised 722 heterosexual participants aged 18–74, mostly women (self-reported gender; 224 men and 498 women) with various educational backgrounds (i.e., High School, Bachelor’s, and Master’s degrees). Participants filled in self-reported scales measuring sexual orientation beliefs (incremental vs. entity views), religiosity, social media use, and perceived gender-transcendent social roles. Correlation analyses and multiple regression models were computed separately for men and women. For all participants, homophobia against lesbians (HAL) was negatively associated with participants’ age, religiosity, and gender-transcendent social roles and positively with incremental views about sexuality. However, only in the case of women was HAL positively related to social media use. Next, for both men and women, homophobia against gay individuals (HAG) was negatively related to age, religiosity, and gender-transcendent social roles. However, only in the case of women, HAG was positively related to social media use online and incremental views about sexuality. In the case of men, the most significant predictor of HAL was the perceived gender-transcendent social roles and HAG – perceived gender-linked social roles. For women, perceived gender-linked social roles were the most significant predictor of both HAL and HAG. Results are discussed regarding their use for interventions aimed at reducing homophobia among heterosexual individuals.

## Introduction

Homophobia persists on the individual and societal levels due to various personal and cultural factors, even though many European countries, including Romania, have become more accepting of sexual diversity. Conceptualized in various ways over time, homophobia generally refers to the fear of being close to homosexuals (Perilli et al., 2021). Homophobia comprises negative attitudes, feelings, or behaviors toward individuals who identify as lesbian, and gay (Madžarević and Soto-Sanfiel, 2018). We consider it important to differentiate two distinct concepts: homophobia and LGBTphobia. Verbal abuse, discrimination, and avoiding interaction are common homophobia manifestations (Herek, 2004). Homophobia can manifest in various ways, including verbal harassment, physical violence, discrimination, and exclusion from social or institutional environments. On the other hand, LGBTphobia refers to rejection, discrimination, and prejudice against all lesbian, gay, bisexual, and transgender (LGBT) individuals (Aguirre et al., 2021).

The negative effects of homophobia on homosexual individuals are highly significant and were the subject of various investigations in this regard. For instance, previous studies suggested a significant relationship between homophobic experiences and mental disorders among sexual minorities (Newcomb and Mustanski, 2010). Moreover, homophobic experiences seem to be related to lower life satisfaction among homosexual individuals (Wen and Zheng, 2019). Furthermore, Poštuvan et al. (2019) suggested that sexual minority youth have a higher risk for suicidal behavior than their heterosexual peers.

Previous research suggested that homophobia is often rooted in social and cultural norms that stigmatize non-heterosexual orientations and gender identities (Meyer, 2013). It was also suggested that these norms are reinforced by religious, political, and educational institutions that promote heteronormativity and gender binary roles (Herek, 2004). Furthermore, the attitudes toward sexual minorities are an important factor in determining the well-being (Poštuvan et al., 2019) and mental health of homosexual people (Padilla et al., 2010). In this regard, various personal, cultural, and social variables were identified in previous studies as protective or risk factors against homophobia. It is also important to mention the minority stress model, based on the social stress theory, which posits that stress can be influenced not only by personal events but also by the social environment (Meyer, 2003) According to this model, discrimination and prejudice rooted in the stigmatization of identity (e.g., race, gender, and sexuality) comprise a powerful and persistent source of stress (Meyer, 1995). Thus, according to the minority stress model, individuals belonging to a minority social group, such as sexual minorities, experience elevated level of stress due to their social position (Meyer, 2003), and this stress that may span multiple generations (Meyer, 2003). Based on this theory, the present study aims to identify factors that can influence homophobia, which can further foster internalized homonegativity among sexual minorities, which, finally, can lead to increased mood and anxiety disorders, health-risk behaviors (e.g., substance use), and risky sexual behaviors (Theodore, 2011).

In the present study, we examined the roles of sexual orientation beliefs, religiosity, perceived social roles, and the social media use, and their roles in explaining homophobia against lesbians and gay individuals among heterosexual adults. More importantly, we focused on these factors among the adults from one of the most religious European states (Dascalu et al., 2021), which recently organized a Referendum to legally “re-define marriage as only being between a man and a woman rather than two spouses” (Maftei and Holman, 2021, p. 305). The Referendum failed to gain validation due to the low turnout of Romanian citizens, which fell short of the required votes to achieve a 30% quorum. As a result, the language in the constitution regarding same-sex marriage remains unchanged, and the possibility of it being legalized in Romania still exists. However, same-sex marriage is not legal in Romania, since the Civil Code’s Article 277 explicitly forbids it (Voiculescu and Groza, 2021). Since previous studies suggested that mental health among LGBTQ+ individuals continues to be lower than average rates (Darwich et al., 2012), and the attitudes toward LGBTQ+ communities are a crucial factor in this regard (Takács, 2015; Maftei and Holman, 2021), we consider it highly important to investigate the protective and risk factors of homophobia among heterosexual Romanian adults.

### Religiosity and homophobia

Religiosity refers to the extent to which individuals hold religious beliefs, engage in religious practices, and find meaning and purpose in religion (Holdcroft, 2006). Previous studies suggested that religiosity can influence individuals’ well-being, with higher religiosity being linked to increased life satisfaction and psychological resilience (Koenig et al., 2012). Moreover, religiosity seems to have a significant influence on social behavior and attitudes in various fields (Donahue and Nielsen, 2005). For instance, several studies suggested that religious individuals might be more likely to engage in pro-social behaviors such as volunteering, donating to charity, and helping others (Roth, 2017). However, religious individuals might also tend to hold negative attitudes toward marginalized groups such as LGBTQ+ individuals or those of other faiths (Westwood, 2022).

The relationship between religiosity and homophobia was a topic of interest to various scholars in the field of social psychology and sociology. For instance, previous studies suggested a positive association between religiosity and homophobia, suggesting that more religious individuals tend to hold negative attitudes toward homosexuality (Van Droogenbroeck et al., 2016).

### Sexual orientation beliefs, perceived social roles, and homophobia

Sexual orientation beliefs refer to the attitudes, beliefs, and values that individuals hold about sexual orientation. These beliefs have significant implications for the way individuals perceive and interact with those who identify as lesbians, gay, bisexual, or queer (Katz-Wise and Hyde, 2015). Previous studies found that individuals who hold negative beliefs about sexual orientation, such as the belief that homosexuality is a sin and morally wrong, might be more likely to hold prejudiced attitudes toward LGBTQ individuals. These individuals view homosexuality as a choice, a mental illness, or a form of deviance, which can lead to feelings of disgust, fear, or anger toward LGBTQ individuals (Murray et al., 2013). On the other hand, individuals who hold positive beliefs about sexual orientation, such as the belief that sexual orientation is a natural aspect of human diversity, might be more likely to hold accepting and supportive attitudes toward LGBTQ individuals. These individuals view homosexuality as a normal variation of human sexuality and support equal rights and protections for LGBTQ communities (Woodford et al., 2012).

In the present study, we focused on Katz-Wise and Hyde’s (2015) view on sexual orientation beliefs, as conceptualized based on Dweck’s et al. (1995) psychological theory of intelligence beliefs and Diamond’s (2008) view of sexual fluidity. More specifically, Katz-Wise and Hyde (2015) reflected on the theory of intelligence (Dweck et al., 1995)—which proposes two views of intelligence beliefs: one view referring to intelligence as being *fixed* or that cannot change (*entity* view), and the other view referring to intelligence as being malleable and can change with effort (*incremental* view). As the authors stated, Dweck’s theory (1995) can also be applied concerning the beliefs about sexual orientation or fluidity: “As applied to sexuality, an entity view suggests that sexuality is fixed and cannot change whereas an incremental view suggests that sexuality is malleable and can change. An incremental view of sexuality may be more consistent with sexual fluidity than an entity view” (Katz-Wise and Hyde, 2015, p. 1461). Moreover, Diamond’s (2008) view of sexual fluidity suggested that sexual orientation is both biological, and environmentally based.

Social roles refer to the social and cultural expectations and norms associated with behaviors, attitudes, and attributes of individuals based on their gender (Eckes and Trautner, 2012). The term “sex” represents a biological construct that refers to the specific attributes in human and animals, and it is related to chromosomes, hormone levels and function, as well as to reproductive or sexual anatomy (Muehlenhard and Peterson, 2011). On the other hand, gender is a multidimensional construct that describes the numerous experiences, roles, and obligations that individuals have, based on their biological sex (Oliffe and Greaves, 2012). Moreover, gender uses biological sex as a foundation to make sense of differences between individuals, classifying them in terms of “women” and “men” as social categories (Muehlenhard and Peterson, 2011). It was suggested that these classifications are social constructs since the vast majority of people identify with one of them (Oliffe and Greaves, 2012). In the present study, we were interested in individuals’ beliefs about gender social roles on two dimensions: gender-linked and gender-transcendent. *Gender-linked roles* are associated with a specific gender, based on the biological sex, while *gender-transcendent roles* are not typically associated with a specific gender (Baber and Tucker, 2006). Previous studies suggested that gender-linked roles generally have negative effects on both men and women, as they limit individuals’ ability to explore different interests and skills (Eagly and Wood, 2012). On the other hand, gender-transcendent roles can promote higher gender equality and might help break down traditional gender barriers (Diekman and Eagly, 2000).

### Social media use: how does the internet shape homophobic attitudes?

In the last decade, social media became an integral part of people’s lives. Millions of users worldwide spend a significant amount of time engaging with online content (Imran, 2014). Moreover, social media platforms transformed the way people communicate, connect, and share information (Olaniran, 2014). Previous studies examined the impact of social media use on various aspects of individuals’ lives, including mental health (Keles et al., 2020), behavior (Singh et al., 2020), and attitudes (Bolton et al., 2013), but the findings in this specific area are mixed. For example, some studies suggested a positive association between social media use and discriminatory attitudes (Ayoub and Garretson, 2017). Moreover, it was found that social media use can predict homophobic attitudes and discrimination against LGBTQ+ individuals (Hu and Li, 2019). Other studies suggested that social media might work as a positive tool for promoting inclusivity and acceptance of sexual and gender minorities (Xie and Peng, 2018). However, Liang et al. (2022) found that the relationship between social media use and attitudes toward homosexuality depends on users’ type, i.e., active (users who use social media for all activities), pragmatic (users who use social media for instrumental purpose), and traditional (individuals who reported lower use of social media). Finally, their results suggested that active and pragmatic users of social media developed more positive views of homosexuality compared to traditional users.

Although some previous studies examined the effect of social media use on homophobic attitudes (e.g., Hu and Li, 2019; Ukonu et al., 2021), there is still a need for empirical evidence regarding the relationship between these variables. Furthermore, previous results are contradictory. Thus, there is still a need to further examine the relationship between social media use and homophobia, and the present study aimed to add some evidence in this regard.

### Age and gender differences regarding homophobia

Some previous studies suggested that younger generations tend to accept homosexuality more and have less homophobic attitudes compared to older generations (Herek and Gonzalez-Rivera, 2006). However, age differences in attitudes toward homosexuality also depend on various cultural and social factors. For example, a study conducted in three Asian cities found that younger people were more homophobic than older people (Feng et al., 2012). Maftei and Holman (2021), on the other hand, suggested that age was not a significant predictor of homophobia in a Romanian sample of young adults.

Gender differences in homophobia have been a topic of interest in psychological research for several decades (Britton, 1990; Reiter, 1991). Previous literature examining the attitudes toward lesbians and gay individuals indicated significant gender-based differences (Yost and Thomas, 2012). Also, some studies suggested that gay men are judged more harshly than lesbians, by both men and women (Fisher et al., 2017). Other scholars suggested that women might be more accepting than men of sexual diversity (Logan, 1996; Yost and Thomas, 2012).

However, researchers also suggested that it is preferable to assess the attitudes toward lesbian and gay individuals separately for men and women because gender has a significant impact in determining attitudes toward sexual diversity (Herek, 2004). It was also suggested that these differences might be linked to traditional gender roles since male homophobic attitudes represent a way to reinforce one’s masculinity (Carnaghi et al., 2011), while traditional gender roles for women emphasize nurturance, caring, and empathy (Rudman and Phelan, 2010). Finally, women’s acceptance of LGBTQ+ individuals might also be related to the traditional women gender roles (Herek, 2004).

### The present study

Given the importance of examining homophobia (due to its significant impact on LGBTQ individuals) and the mixed findings regarding the role of age, gender, religiosity, sexual orientation beliefs, social roles beliefs, and social media use in this regard, the present study aimed to examine these possible predictors of homophobia in a Romanian sample of heterosexual adults.

Based on previous research, in the present study, we focused on the predictive roles of age (Herek and Gonzalez-Rivera, 2006), sexual orientation beliefs (Katz-Wise and Hyde, 2015), social roles beliefs (Eagly and Wood, 2012), religiosity (Van Droogenbroeck et al., 2016), and social media use (Hu and Li, 2019). These factors were not simultaneously examined as predictors of homophobia, shaping one of the present research’s most important contributions. Furthermore, we also aimed to examine these predictors separately, for men and women participants, and to refer to two specific forms of homophobia, i.e., homophobia against lesbians and homophobia against gay individuals.

## Method

### Participants and procedure

Our sample comprised 722 heterosexual participants aged 18 to 74 (*M* = 27.62, SD = 9.18), mostly women (self-reported gender, open question; 224 men, and 498 women), with various educational backgrounds (i.e., High School – 35.7%, *N* = 257; Bachelor’s degree – *N* = 355, 49.2%, and Master’s degree – *N* = 110, 15.1%). More specifically, 86 men (38.3%), and 171 women (34.3%) reported their high school educational level, 80 men (35.7%), and 275 women (55.2%) reported their Bachelor’s educational level, and 58 men (25.9%), and 52 women (10.4%) reported their Master’s educational level. Participants added their answers to a web-based survey. We performed a sensitivity power analysis using G*Power 3.1 (Faul et al., 2007) to identify the minimum sample size needed in the research design that we used. For a medium effect f2 = 0.15, with an alpha = 0.05 and power = 0.95, the minimum sample required was 146.

The study was advertised through social media platforms (i.e., Facebook and Instagram), and official and informal groups. The only included criterion was related to age (>18) and sexual orientation (i.e., heterosexual). Before beginning the survey, participants were given an informed consent form indicating the study’s goals, the estimated time of their participation (about 15 min), the confidentiality and anonymity of their responses, and the option to refuse or quit the study at any point. The current study, which followed the 2013 Helsinki Declaration’s ethical principles, was approved by The Ethics Committee of the Faculty, where the authors are affiliated.

### Measures

#### Sexual orientation beliefs

We used the 8-item Entity vs. Incremental Views of Sexual Orientation scale (Katz-Wise and Hyde, 2015) to measure participants’ sexual orientation beliefs. Items were measured on a 7-point Likert scale ranging from 1 (*strongly disagree*) to 7 (*strongly agree*). Higher scores indicated an *incremental* view, that sexuality can change or a person can change their sexuality, and lower scores indicating an *entity* view, that sexuality is fixed or a person cannot change their sexuality. Example items included “A person’s sexual orientation is something very basic about them that they cannot change” (entity view of sexuality), and “A person can substantially change their sexual orientation” (incremental view of sexuality). In the present study, Cronbach’s alpha was 0.71 for the men’s subsample, and 0.74 for the women’s subsample. The scale was used in previous similar studies, demonstrating good psychometric properties (e.g., Hammack et al., 2019).

#### Religiosity

We used the Romanian version of the Centrality of Religiosity Scale (CRS 15; Gheorghe, 2018) to measure participants’ religiosity. Participants answered on a 5-point Likert scale ranging from 1 (*never*) to 5 (*always*). Example items included “How often do you think about religious issues?,” and “How often do you pray?.” In the present study, Cronbach’s alpha was 0.91 for the men’s subsample, and 0.92 for the women’s subsample. As in previous studies (e.g., Ackert and Plopeanu, 2020), we used the scale’s total score, with higher scores indicating higher religiosity.

#### Social roles beliefs

We used the Social Roles Questionnaire (Baber and Tucker, 2006) to measure participants’ attitudes toward gender role stereotyping. Items were measured on a 5-point Likert scale ranging from 1 (*strongly* disagree) to 5 (*strongly* agree). The Social Roles Questionnaire contains two subscales, i.e., *gender transcendent*, and *gender-linked* subscales, with higher scores indicating negative attitudes toward gender role stereotyping. Example items included “People should be treated the same regardless of their sex” (gender transcendent), and “Some types of work are just not appropriate for women” (gender-linked). In the present study, Cronbach’s alpha was 0.70 for the men’s subsample, and 0.69 for the women’s subsample for the gender-transcendent subscale and 0.78 for the men’s subsample, and 0.76 for the women’s subsample for the gender-linked subscale. The scale was used in previous similar studies, demonstrating good psychometric properties (Davies et al., 2012).

#### Social media use

We used the Social Media Use Integration Scale (Jenkins-Guarnieri et al., 2013) to evaluate participants’ social media use. Items were measured on a 6-point Likert scale ranging from 1 (*strongly disagree*) to 6 (*strongly agree*). The SMUI contains two subscales, i.e., the Social Integration and Emotional Connection, and the Integration into Social Routines subscales. Example items included “I feel disconnected from friends when I have not logged into Facebook” (the social integration and emotional connection), and “Using Facebook is part of my everyday routine” (the integration into social routines). Higher scores indicated more engaged use and integration of social media. In the present study, Cronbach’s alpha for the Social Integration and Emotional Connection subscale was 0.89 for the men’s subsample and 0.88 for the women’s subsample, and for the Integration into Social Routines subscale was 0.63 for the men’s subsample, and 0.69 for the women’s subsample, and overall Cronbach’s alpha was 0.88. The scale was used in previous studies, demonstrating good psychometric properties (e.g., Berryman et al., 2018).

#### Homophobia

We used the Modern Homophobia Scale (Raja, 1998) to evaluate participants’ homophobia. Items were measured on a 5-point Likert scale ranging from 1 (*strongly disagree*) to 5 (*strongly agree*). The scale contains two subscales, i.e., modern homophobia–lesbians, and modern homophobia—gay scales. Example items included “I would not mind working with a lesbian” (homophobia against lesbians) and “It’s all right to with me if I see two men holding hands” (homophobia against gay individuals). Higher scores indicated a high level of homophobia for each dimension (lesbian or gay). In the present study, Cronbach’s alpha was 0.91 for the men’s subsample and 0.92 for the women’s subsample for the homophobia toward lesbians subscale and 0.95 for the men’s subsample and 0.96 for the women’s subsample for the homophobia toward gay individuals subscale. The instrument was used in previous studies that demonstrated its good psychometric properties (e.g., Fisher et al., 2017).

#### Demographics

Finally, a demographic scale assessed participants’ age, self-reported gender, and educational level.

The consistency of the quality of the translated research instruments (which were presented in Romanian) was checked using the back-translation technique (Tyupa, 2011), and no discrepancies were identified.

#### Overview of the statistical analyses

We used the 26 version of the IBM SPSS statistical package to analyze our data. All participants filled in all the answers, so our data had no missing values. We first conducted zero-order bivariate correlations between the study’s main variables, separately for men and women participants. Next, we conducted multiple regression models separately for men and women participants.

## Results

The descriptive statistics (i.e., means, standard deviations, minimum, and maximum scores) for the primary variables for each subsample (i.e., men and women) are presented in Tables 1, 2. Normally distributed variables should have Skewness between −1 and 1 and Kurtosis between −3 and 3 (Kim, 2013), and this was the case for all the variables in the present study.

**Table 1:** Descriptives statistics for the primary variables (men sample; *N* = 224).

	*M*	SD	Min	Max	Skewness	Kurtosis
1. Age	28.05	8.88	18	72	–	–
2. Sexual orientation beliefs	15.82	3.80	4	28	−0.01	1.08
3. Perceived gender-transcendent social roles	12.62	4.08	5	25	0.02	−0.44
4. Perceived gender-linked social roles	24.48	5.90	8	40	0.06	0.00
5. Religiosity	41.26	11.87	15	74	0.23	0.03
6. Social media use	31.88	9.62	10	60	0.21	0.03
7. Homophobia against lesbians	75.86	17.18	32	115	0.27	0.34
8. Homophobia against gay individuals	66.73	18.19	22	110	0.07	0.83

**Table 2:** Descriptives statistics for the primary variables (women sample; *N* = 498).

	*M*	SD	Min	Max	Skewness	Kurtosis
1. Age	27.43	9.31	18	74	–	–
2. Sexual orientation beliefs	16.96	4.23	4	28	−0.30	−0.12
3. Perceived gender-transcendent social roles	10.34	3.25	5	22	0.64	0.26
4. Perceived gender-linked social roles	23.78	6.35	8	40	−0.08	−0.09
5. Religiosity	48.06	12.65	15	75	−0.32	−0.22
6. Social media use	33.81	10.47	10	60	−0.05	−0.20
7. Homophobia against lesbians	82.96	21.57	28	120	−0.15	−0.69
8. Homophobia against gay individuals	76.47	22.61	22	110	−0.14	−0.78

For the men’s group, the Pearson correlation analysis indicated significant negative associations between age and homophobia, both toward lesbians (*r* = −0.18; *p* = 0.005) and gay (*r* = −0.13; *p* = 0.03). Thus, the higher the men’s age, the higher the homophobia toward lesbians and gay individuals. Moreover, results indicated significant negative relationships between social roles beliefs, and homophobia against lesbians and gay. More specifically, results indicated a significant negative relationship between gender-transcendent social roles and homophobia, both toward lesbians (*r* = − 0.35; *p* < 0.001) and gay individuals (*r* = −0.17; *p* = 0.009), as well as between gender-linked social roles and homophobia, both toward lesbians (*r* = −0.19; *p* = 0.003) and gay individuals (*r* = −0.341; *p* < 0.001). Moreover, results suggested a significant negative association between religiosity and homophobia, both toward lesbians (*r* = −0.26; *p* < 0.001) and gay individuals (*r* = −0.35; *p* < 0.001). The only positive correlation was found between homophobia against lesbians and sexual orientation beliefs (*r* = 0.19; *p* = 0.003). However, our results indicated no significant relationships between social media use and homophobia against lesbians or gay among men (see Table 3).

**Table 3:** Zero-order correlations among the primary variables (men sample; *N* = 224).

	1	2	3	4	5	6	7	8
1. Age	–							
2. Sexual orientation beliefs	−0.07	–						
3. Perceived gender-transcendent social roles	−0.00	−0.28**	–					
4. Perceived gender-linked social roles	−0.01	0.17**	−0.33**	–				
5. Religiosity	−0.04	0.11	−0.08	0.31**	–			
6. Social media use	−0.11	0.01	−0.03	0.06	0.04	–		
7. Homophobia against lesbians	−0.18**	0.19	−0.35**	−0.19**	0.26**	−0.01	–	
8. Homophobia against gay	−0.13**	0.06	−0.17**	−0.34**	−0.35**	−0.05	0.82**	–

**p < 0.001.

In the women’s’ group, the Pearson correlation analysis indicated significant and negative associations between age and homophobia, both toward lesbians (*r* = −0.44; *p* = 0.001) and gay individuals (*r* = −0.14; *p* = 0.002). Thus, the higher the age, the higher the homophobia. Moreover, data suggested significant negative relationships between social roles beliefs, and homophobia against both lesbians and gay individuals. More specifically, we found a significant negative relationship between gender-transcendent social roles and homophobia, both toward lesbians (*r* = −0.31; *p* < 0.001) and gay individuals (*r* = −0.300; *p* < 0.001), as well as between gender-linked social roles and homophobia, both toward lesbians (*r* = −0.43; *p* < 0.001) and gay individuals (*r* = −0.48; *p* < 0.001). Also, results suggested a significant negative association between religiosity and homophobia, both toward lesbians (*r* = −0.45; *p* < 0.001) and gay individuals (*r* = −0.44; *p* < 0.001). Results also indicated positive relationships between sexual orientation beliefs and homophobia, both toward lesbians (*r* = 0.17; *p* < 0.001) and gay individuals (*r* = 0.14; *p* = 0.001). However, results indicated no significant relationships between social media use and homophobia against lesbians and gay among women (all *p*-s > 0.05) (see Table 4).

**Table 4:** Zero-order correlations among the primary variables (women sample; *N* = 498).

	1	2	3	4	5	6	7	8
1. Age	–							
2. Sexual orientation beliefs	0.05	–						
3. Perceived gender-transcendent social roles	−0.03	−0.10*	–					
4. Perceived gender-linked social roles	0.10*	0.03	0.05	–				
5. Religiosity	0.05	−0.11**	0.11*	0.32**	–			
6. Social media use	0.03	0.08	−0.05	0.05	−0.02	–		
7. Homophobia against lesbians	−0.14**	0.17**	−0.31**	−0.43**	−0.45**	0.02	–	
8. Homophobia against gay	−0.14**	0.14**	−0.30**	−0.48**	−0.44**	0.00	0.93**	–

**p < 0.001.

*p < 0.05.

Based on correlation results, we further conducted multiple regression analyses for each group of participants (i.e., men and women) with homophobia scores as the dependent variables. We were interested to examine whether age, sexual orientation beliefs, social roles beliefs, religiosity, and social media use significantly predicted homophobia. The multiple regression models were computed separately for men and women, regarding homophobia toward both lesbians and gay individuals.

### Regression analyses—men sample

In the men’s group, sexual orientation beliefs, gender transcendent, gender-linked, and religiosity were significant predictors of homophobia against lesbians (HAL). All independent variables that were included in the regression model (i.e., age, religiosity, sexual orientation beliefs, social roles beliefs, and social media use) explained 33.1% of the variation in homophobia against lesbians, *F* (6, 223) = 17.91, *p* < 0.001. The most significant predictor of HAL was the perceived gender-transcendent social roles (β = 0.42, *p* < 0.001). Age, perceived gender-transcendent social roles, perceived gender-linked social roles, and religiosity were significant predictors of homophobia against gay individuals (HAG). Thus, the higher perceived gender-linked social roles, and religiosity, the higher homophobia against gay individuals. Also, the higher perceived gender-transcendent social roles, the lower homophobia against gay individuals. All independent variables included in the regression model (i.e., age, religiosity, sexual orientation beliefs, social roles beliefs, and social media use) explained 30.2% of the variation in homophobia against gay individuals, *F* (6, 223) = 15.67, *p* < 0.001. The most significant predictor of HAG was the perceived gender-linked social roles (β = 0.36, *p* < 0.001) (see Table 5).

**Table 5:** Summary of linear regression analysis for the attitude toward homosexuality (men sample; *N* = 224).

	HAL			HAG		
Variables	*B*	SE (*B*)	*β*	*B*	SE (*B*)	*β*
Age	−0.37**	0.10	−0.19	−0.30*	0.11	−0.15
Sexual orientation beliefs	0.61*	0.26	0.13	0.29	0.28	0.06
Perceived gender-transcendent social roles	−1.80**	0.25	−0.42	−1.35**	0.27	−0.30
Perceived gender-linked social roles	−0.84**	0.18	−0.29	−1.12**	0.19	−0.36
Religiosity	−0.33**	0.08	−0.23	−0.42**	0.09	−0.27
Social media use	0.03	0.10	0.62	−0.03	0.10	−0.01
*R*^2^	0.33**			0.30**		

*p < 0.05.

**p < 0.001.

### Regression analyses—women sample

In the women group, age, sexual orientation beliefs, gender transcendent, gender-linked, and religiosity were significant predictors of HAL. The independent variables that were included in the regression model explained 39.2% of the variation in HAL, *F* (6, 497) = 52.73, *p* < 0.001. The most significant predictor of HAL was perceived gender-linked social roles (β = 0.31, *p* < 0.001). Further, age, sexual orientation beliefs, gender transcendent, gender-linked, and religiosity was a significant predictor of HAG. Thus, the higher age, sexual orientation beliefs, perceived gender-linked social roles, and religiosity, the higher homophobia against lesbians. Also, the higher and gender transcendent social roles, the lower homophobia against lesbians. The proposed variables explained 40.4% of the variation in HAG, *F* (6, 497) = 55.39, *p* < 0.001. The most significant predictor of HAG was perceived gender-linked social roles (β = 0.37, *p* < 0.001) (see Table 6).

**Table 6:** Summary of linear regression analysis for the attitude toward homosexuality among (women sample; *N* = 498).

	HAL			HAG		
Variables	*B*	SE (*B*)	*β*	*B*	SE (*B*)	*β*
Age	−0.25*	0.08	−0.11	−0.24*	0.08	−0.10
Sexual orientation beliefs	0.65**	0.18	0.12	0.57*	0.19	0.10
Perceived gender-transcendent social roles	−1.66**	0.23	−0.25	−1.66**	0.24	−0.24
Perceived gender-linked social roles	−1.06**	0.12	−0.31	−1.33**	0.13	−0.37
Religiosity	−0.52**	0.06	−0.30	−0.49**	0.06	−0.27
Social media use	−0.02	0.07	0.01	−0.01	0.07	−0.00
*R*^2^	0.39**			0.40**		

*p < 0.05.

**p < 0.001.

## Discussion

The present study aimed to examine some potential predictors of homophobia against lesbians and gay individuals. Previous research found that age (Herek and Gonzalez-Rivera, 2006), gender (Yost and Thomas, 2012), religiosity (Van Droogenbroeck et al., 2016), sexual orientation beliefs (Murray et al., 2013), social roles beliefs (Eagly and Wood, 2012), and social media use (Hu and Li, 2019) are significant predictors of general homophobia. We analyzed these predictors together and computed a linear regression model for each type of homophobia. Moreover, we examined the regression model separately for men and women participants.

Our results suggested that the proposed regression models were significant, explaining 33.1% of the variation in homophobia against lesbians and 30.2% of the variation in homophobia against gay individuals in the men participants’ group. Furthermore, the regression model explained 39.2% of the variation in homophobia against lesbians and 40.4% of the variation in homophobia against gay individuals in the women participants’ group. Therefore, our results align with previous studies which suggested a significant relationship between age and homophobic attitudes (Herek and Gonzalez-Rivera, 2006). Thus, the higher individuals’ age, the higher level of homophobic attitudes. Our results indicated that younger individuals might hold less homophobic attitudes compared to older ones.

In line with previous studies (e.g., Bengtson et al., 2015), our results suggested that both age and religiosity were significant predictors of homophobia for both men and women participants. Also, our results align with previous findings that suggested a positive correlation between religiosity and negative attitudes toward homosexuality (Van Droogenbroeck et al., 2016). One explanation for this relationship is that certain religious beliefs might promote negative attitudes toward homosexuality, based on the view that homosexuality is a sin (Woodford et al., 2021). It is also important to mention that the relationship between religiosity and negative attitudes toward homosexuality was previously examined based on religious affiliations. For example, some studies suggested that individuals belonging to Conservative Protestant denominations might hold the most unfavorable attitudes toward homosexuals, followed by moderate Protestants and Catholics (Maher, 2013). Other studies suggested that Orthodox Jews might hold more prejudice toward homosexuals than Progressive Jews (Roggemans et al., 2015). However, although most religions condemn certain forms of discrimination, such as racial or ethnic prejudice, they allow other forms of prejudice toward individuals who are perceived to contravene the religion’s value system. As a result, many religious groups regard lesbians and gay individuals as falling into this category (Roggemans et al., 2015). Based on these findings, we argue that future studies might benefit from additionally examining the influence of religious affiliations on homophobic attitudes.

The present findings also indicated that sexual orientation beliefs and social role beliefs were significant predictors of homophobia against lesbians and gay individuals. As in previous studies, participants who believed that sexual orientation was a choice reported more negative attitudes toward homosexuality than those who viewed sexual orientation as a natural aspect of human diversity (Woodford et al., 2012). Regarding social role beliefs, our results revealed that both gender-transcendent and gender-linked social roles were significant predictors of homophobia. Although there is a lack of empirical evidence toward the relationship between gender-transcendent, gender-linked social roles, and homophobia, we argue that these results may be explained through the association between gender-linked and specific gender social roles. Thus, individuals’ beliefs about gender roles and obligations might lead to more homophobic attitudes, based on the idea that some specific social roles are better suited for either men or women (Baber and Tucker, 2006).

Although previous studies regarding the relationship between social media use and homophobic attitudes are contradictory (e.g., Xie and Peng, 2018; Hu and Li, 2019), our results did not reveal a significant relationship between social media use and homophobia. One possible explanation for these results could pertain to the content of social media. Social networks depend on an algorithm that customizes content for each user. As a result, individuals who disregard posts related to homosexuality or gender diversity may not be exposed to this type of content (Bakshy et al., 2015). Therefore, we believe that future studies might benefit from experimentally investigating the effect of social media usage on homophobic attitudes, with emphasis on the type of content that participants are exposed to.

There are several theoretical and practical implications of the present findings. From a theoretical point of view, these findings contribute to the growing body of research aimed to understand the protective and risk factors associated with homophobic attitudes. Understanding the factors that contribute to the development and maintenance of negative attitudes toward the LGBTQ+ community is essential for developing effective interventions aimed to reduce homophobia, such as culturally sensitive therapy, anti-discrimination laws, inclusive education, policy development, and advocacy efforts to create a more inclusive and accepting society. The present study’s identification of age, religiosity, sexual orientation beliefs, and gender social roles as significant predictors of homophobia provides a framework for future research to explore these underlying mechanisms further, especially in high religious countries, such as Romania. Further, to better understand the associations between the proposed variables, and the insights related to gender-based differences, future studies might benefit from computing statistical comparisons of correlation coefficients- e.g., using Fisher’s r to z transformation.

Moreover, future research might use other methods (e.g., qualitative, longitudinal, or experimental designs) to uncover the underlying cognitive, social, and cultural processes in developing homophobia. Also, further studies might explore the perceptions of individuals belonging to sexual minorities regarding homophobia, emphasizing the social and situational context in which they have encountered homophobic attitudes. Furthermore, future extension of the present results might also include other gender or sexual minorities (Salvati and Koc, 2022), and the examination of sexual prejudice in different social contexts (De Cristofaro et al., 2020; Salvati et al., 2020). Nevertheless, the challenges of conducting a study involving diverse sexual participants are linked to their reticence to participate, as well as the fear of not disclosing their identity, especially in countries such as Romania. Nevertheless, the present findings might contribute to the development of targeted interventions (e.g., education and contact with LGBTQ people, legal awareness initiatives) aimed to reduce the negative attitudes toward LGBTQ+ individuals.

Finally, several limitations need to be addressed for the present study. First, our sample size was relatively small. Future research on this topic might explore these variables in larger, and more diverse groups of participants. Additionally, convenient sampling has the disadvantage of reducing the generalizability of our findings. Second, we measured the study’s variables in a self-reported way that may have increased the likelihood of desirable answers. Alternative measurements might be used in future studies, such as experimental approaches, to reduce this risk. Third, the sample we used was rather unbalanced in terms of gender. Future studies might benefit from using more gender-balanced samples. Also, the generalizability of the present findings is reduced to heterosexual individuals only.

## Conclusion

In conclusion, the present study provides important insights related to the factors that contribute to homophobia against lesbians and gay individuals. The findings suggested that age, religiosity, sexual orientation beliefs, and social role beliefs were significant predictors of homophobic attitudes. Overall, this study highlights the importance of promoting equality, diversity, and inclusivity, as well as working toward reducing discrimination and prejudice against marginalized groups, including the LGBTQ+ community.

## Data availability statement

The raw data supporting the conclusions of this article will be made available by the authors, without undue reservation.

## Ethics statement

The studies involving human participants were reviewed and approved by Faculty of Psychology and Education Sciences, “Alexandru Ioan Cuza” University (Iasi, Romania). The patients/participants provided their written informed consent to participate in this study.

## Author contributions

All authors listed have made a substantial, direct, and intellectual contribution to the work and approved it for publication.

## Conflict of interest

The authors declare that the research was conducted in the absence of any commercial or financial relationships that could be construed as a potential conflict of interest.

## Publisher’s note

All claims expressed in this article are solely those of the authors and do not necessarily represent those of their affiliated organizations, or those of the publisher, the editors and the reviewers. Any product that may be evaluated in this article, or claim that may be made by its manufacturer, is not guaranteed or endorsed by the publisher.
